# Human Milk Oligosaccharides in the Prevention of Necrotizing Enterocolitis: A Journey From *in vitro* and *in vivo* Models to Mother-Infant Cohort Studies

**DOI:** 10.3389/fped.2018.00385

**Published:** 2018-12-04

**Authors:** Lars Bode

**Affiliations:** Department of Pediatrics and Larsson-Rosenquist Foundation Mother-Milk-Infant Center of Research Excellence (LRF MOMI CORE), University of California, San Diego, La Jolla, CA, United States

**Keywords:** preterm infant, necrotizing enterocolitis, breast milk, infant nutrition, human milk oligosaccharide

## Abstract

Preterm infants who receive human milk instead of formula are 6- to 10-times less likely to develop necrotizing enterocolitis (NEC), one of the most common and devastating intestinal disorders that affects 5–10% of all very-low-birth-weight infants. Combined data from *in vitro* tissue culture models, *in vivo* preclinical studies in animal models, as well human mother-infant cohort studies support the hypothesis that human milk oligosaccharides (HMOs), complex sugars that are highly abundant in human milk but not in infant formula, contribute to the beneficial effects of human milk feeding in reducing NEC. The almost 20-year long journey of testing this hypothesis took an interesting turn during HMO *in vivo* efficacy testing and structure elucidation, suggesting that the original hypothesis may indeed be correct and specific HMO reduce NEC risk, however, the underlying mechanisms are likely different than originally postulated.

## Necrotizing Enterocolitis (NEC) Is One of the Most Common and Devastating Intestinal Disorders in Preterm Infants, But Therapeutic Options Are Limited

Necrotizing enterocolitis (NEC) is one of the most common and devastating intestinal disorders in preterm infants [reviewed in (1)]. In the United States and Canada the mean prevalence of NEC in infants with a birth weight between 500 and 1,500 g is about 7%, but can be much higher in certain neonatal intensive care units (NICUs) (2–5). NEC is one the most common causes of gastrointestinal surgical emergencies among neonates and is also the most common cause of death among neonates requiring gastrointestinal surgery (6–8). The mortality rate for NEC patients ranges from 10 to 50% and approaches 100% for patients with the most severe form of the disease (2). Survivors are often faced with long-term neurological complications (9). The total annual costs to care for infants with NEC in the United States alone are estimated to be between $500 million and $1 billion (1, 10, 11).

Medical interventions to treat NEC are limited and typically include bowel decompression, discontinuation of enteral feeding and broad-spectrum intravenous antibiotics [reviewed in (1, 12, 13)]. Surgical interventions range from drain placement to resection of diseased bowel, but once surgery is required, the outcome is often poor.

The rapid onset, fulminant progression, and limited treatment options make it most desirable to prevent NEC all together before it strikes. Preventative approaches include the use of enteral antibiotics, administering pre-, pro-, or synbiotics, growth factors, cytokines, and glucocorticoids [reviewed in (1, 14, 15)]. Most of these approaches are however controversial (16–18) or have not been validated in preclinical or clinical studies.

Overall, therapeutic options to treat or prevent NEC are highly limited. New safe and effective NEC therapies are urgently needed to meet the clinical needs of preterm infants that suffer from this devastating condition.

## NEC Etiology And Pathogenesis Are Complex And in Part Undefined

**Figure 1:**
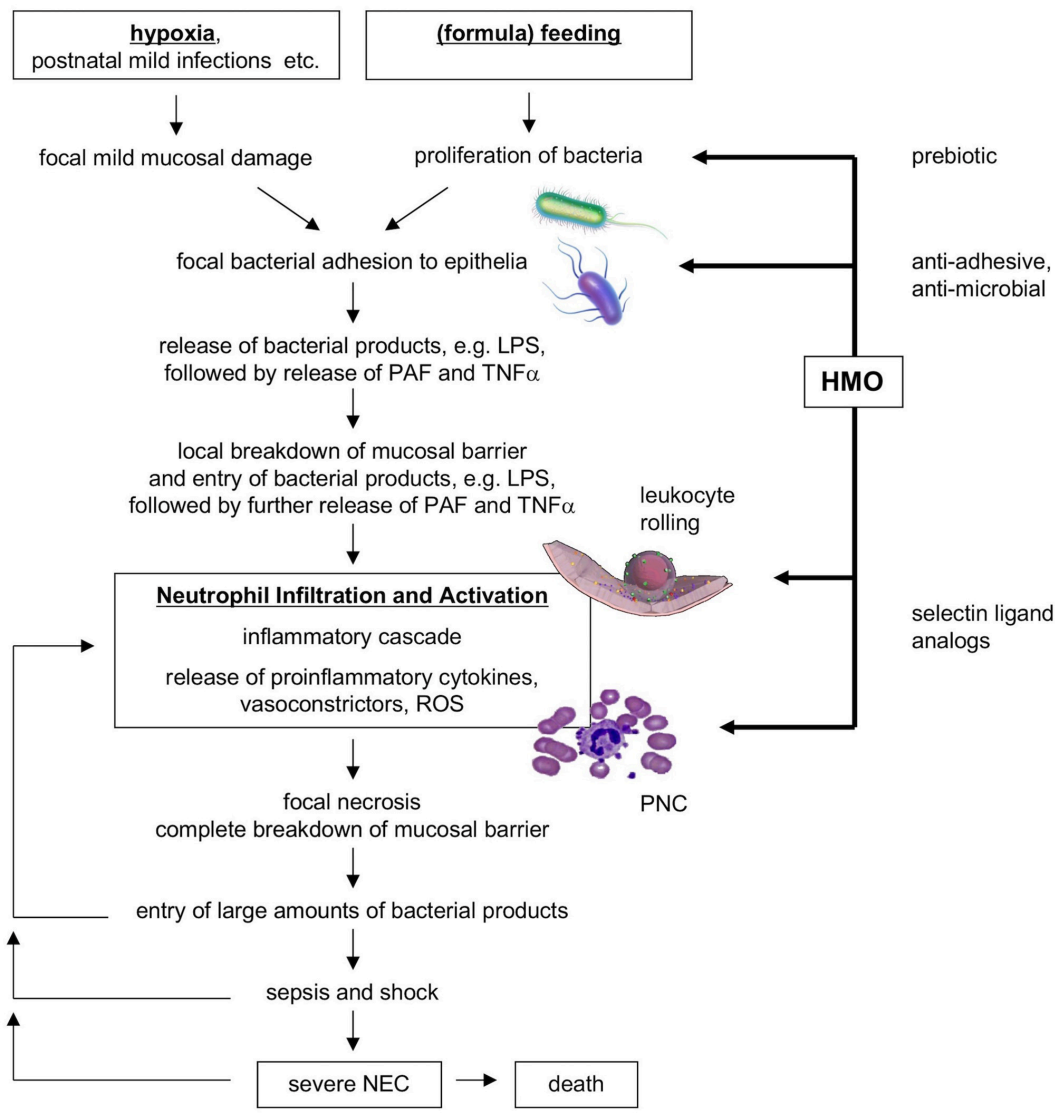
Flow diagram of proposed NEC pathogenesis and potential benefits of HMOs. HMO, human milk oligosaccharides; NEC, necrotizing enterocolitis; PAF, platelet-activating factor; PNC, platelet-neutrophil complexes; ROS, reactive oxygen species [modified after Hsueh et al. (19)].

Instead of representing one clearly defined disorder, NEC may represent a syndrome, with a variety of etiologies and commonalities in the underlying pathogenetic mechanisms. Although NEC pathogenesis is incompletely understood, it likely involves intestinal immaturity and an excessive inflammatory response to an imbalance in the microbial colonization of the infant's intestine [dysbiosis; reviewed in (1, 19)]. One of the proposed models suggests that perinatal hypoxia or a mild postnatal infection could be the primary insults causing mild mucosal damage and impaired intestinal epithelial barrier function (19). Following (formula) feeding and a proliferation of the intestinal microbiome, an increased uptake of bacteria and bacterial metabolites including lipopolysaccharides (LPS) into the mucosa triggers the endogenous production of inflammatory cytokines such as platelet-activating factor (PAF) and tumor necrosis factor alpha (TNFα), which in turn further enhance intestinal permeability, closing a vicious circle. PAF also synergizes with LPS and TNFα, reaching a threshold necessary to induce an inflammatory cascade, which includes mucosal neutrophil infiltration and activation. Eventually, vasoconstriction occurs and leads to ischemia and subsequent reperfusion. Reactive oxygen species (ROS) produced by activated neutrophils and intestinal epithelial xanthine oxidase may then cause severe tissue necrosis and breakdown of the intestinal barrier. Entry of large amounts of bacteria and LPS leads to sepsis, shock, and death. Figure 1 shows a flow diagram of the proposed pathogenesis of NEC [modified after Hsueh et al. (19)].

## NEC Incidence Is Significantly Lower in Human Milk-Fed Infants Compared to Formula-Fed Infants

Several studies have shown that NEC incidence is 6- to 10-fold lower in human milk-fed infants compared to formula-fed infants (20–23). It is unclear whether components in infant formula trigger NEC, whether components in human milk protect from NEC, or whether a combination of both is responsible for the gap in NEC incidence between human milk-fed and formula-fed infants. However, a significant number of infants still develop NEC although they exclusively receive human milk and are not exposed to infant formula. These observations speak against the notion that components in infant formula trigger NEC and support the idea that bioactive components in human milk protect from NEC. Interpersonal variation in human milk composition may explain why some infants still develop NEC despite receiving human milk.

## Human Milk Oligosaccharides (HMOs) Are the Third Most Abundant Component of Human Milk. HMOs Help Shape the Infant Gut Microbiome and May Prevent NEC-Associated Dysbiosis

Human milk contains a high amount of complex glycans (carbohydrates, sugars) that are not present in infant formula [reviewed in (24–28)], which led us to hypothesize that these human milk oligosaccharides (HMOs) at least in part contribute to the lower incidence of NEC in human milk-fed infants. HMOs are the third most abundant component in human milk (5–20 g/L), surpassed only by the concentrations of lactose (~70 g/L) and lipids (~40 g/L). HMO composition follows a basic blueprint that connects the five building blocks glucose (Glc), galactose (Gal), N-acetylglucosamine (GlcNAc), fucose (Fuc), and the sialic acid derivative N-acetylneuraminic acid (Neu5Ac) in specific linkages. More than 150 different and structurally distinct HMOs have been identified and the composition varies between women as well as over the course of lactation. Once ingested, HMOs withstand the low pH in the infant's stomach (29), resist degradation by pancreatic and brush border membrane enzymes (29, 30), and reach the infant's distal small intestine and colon. Here, HMOs act as prebiotics that serve as metabolic substrates for potentially beneficial bacteria to thrive while supressing other, potentially harmful bacteria. In addition, HMOs are antiadhesives that serve as soluble adhesion receptor decoys and block the attachment of potential viral or bacterial pathogens to the infant's intestinal epithelial cell surface, a process that otherwise allows pathogens to proliferate, and in some cases invade, and cause disease. Moreover, HMOs are antimicrobials that directly kill bacteria (cytotoxic) or at least reduce bacterial proliferation (cytostatic). Altogether, these mechanisms shape the infant gut microbiome early in life (26–28, 31) and may counteract dysbiosis at the early stages of NEC pathogenesis shown in Figure 1.

## HMOs Are Absorbed Intact, Interfere With Immune Cell-Cell Interactions, and May Reduce NEC-Associated Inflammation

HMOs are not only present in the infant's intestinal lumen, they are also absorbed, reach the systemic circulation, and are excreted intact with the infant's urine (32–35). Thus, in addition to effects in the intestinal lumen, HMOs may also interfere with the inflammatory cascade involved in NEC pathogenesis.

**Figure 2:**
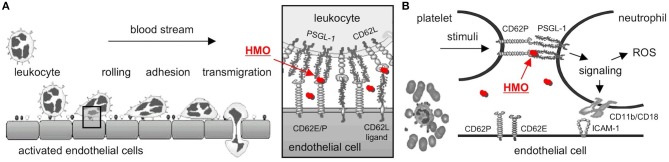
Selectin-mediated cell-cell interactions and potential interference with HMOs. **(A)** Leukocytes decelerate from the blood stream before they adhere and finally transmigrate to the site of inflammation. The initial rolling, the first interaction between leukocytes and activated endothelial cells, is mediated by selectins (box). HMOs (red dots) serve as selectin ligand analogs, reduce selectin ligand binding, and are thought to reduce leukocyte rolling and infiltration (modified after http://ley-leukocyte.liai.org). **(B)** Activated platelets upregulate expression of P-selectin (CD62P), which binds to P-Selectin Glycoprotein Ligand-1 (PSGL-1) on neutrophils, which establishes platelet-neutrophil-complex (PNC) formation and triggers a signaling cascade with an increase in neutrophil adhesion molecules and production of reactive oxygen species (ROS). Once again, HMOs serve as selectin-ligand analogs, reduce P-selectin-PSGL-1 binding, and neutrophil activation [modified after Cerletti et al. (36)]. CD62E/P, E- or P-Selectin; CD62L, L-Selectin; HMO, human milk oligosaccharide; PSGL-1, P-Selectin Glycoprotein Ligand-1.

Mucosal neutrophil infiltration and activation are thought to be early key events in NEC pathogenesis. Neutrophils are first decelerated from the blood stream before they adhere to endothelial cells and transmigrate (Figure 2A). Neutrophil deceleration, the “rolling” on activated endothelial cells, is mediated by adhesion molecules of the selectin family (37). Selectins bind to carbohydrate determinants, predominantly Sialyl Lewis x (SLe^x^; NeuAcα2-3Galβ1-4(Fucα1-3)GalNAc) (38), on glycoconjugate ligands. While L-selectin (CD62L) is constitutively expressed on leukocytes, P- and E-selectins (CD62P and E) are expressed on platelets and endothelial cells, and their expression is upregulated by inflammatory cytokines such as TNFα. P-selectin expression is increased on intestinal endothelial cells in NEC patients and strongly correlates with neutrophil infiltration (39). P-selectin knockout mice are protected from PAF-induced intestinal necrosis (40), indicating an essential role of P-selectin in NEC pathogenesis.

Neutrophil activation and ROS production lead to progression of NEC pathogenesis. Platelet-neutrophil complexes (PNC) represent a highly activated subpopulation of neutrophils primed for adhesion and increased ROS production. PNC formation is increased after ischemia/reperfusion (41, 42), one of the key events in NEC progression. PNC formation is initiated by the binding of P-selectin on activated platelets to PSGL-1 on neutrophils (Figure 2B). PSGL-1 signaling induces ROS production as well as expression of CD11b/CD18 adhesion molecules that facilitate neutrophil binding to platelets and activated endothelial cells (36). Blocking P-selectin with a P-selectin antibody completely inhibits the increase in ROS production and CD11b/CD18 expression (43), documenting the essential role of P-selectin in PNC formation.

While both neutrophil infiltration as well as neutrophil activation and ROS production require selectin-ligand interactions, HMOs have been shown to carry SLex determinants, suggesting they act as soluble selectin ligand analogs (44). Indeed, we were the first to provide evidence that a mixture of sialylated HMOs reduces selectin-mediated neutrophil rolling and adhesion *in vitro* (45) as well as PNC formation and neutrophil activation *ex vivo* (46). However, at this stage it was unclear whether or not these *in vitro*/*ex vivo* results translate to *in vivo*, inhibit key events in NEC pathogenesis, and reduce or prevent NEC.

## *In vivo* Efficacy Testing in a Neonatal Rat Model Confirms That HMOs Reduce NEC-Like Symptoms and Improve Survival

Results from *in vitro* and *ex vivo* studies supported our hypothesis that HMOs contribute to a lower NEC risk in human milk-fed infants, but to confirm this hypothesis, we needed *in vivo* proof—ideally by showing HMO efficacy in preterm infants. However, at this stage, a human intervention study was not feasible for several reasons: (1) We would need to recruit between 800 and 1,000 preterm infants to power the study. (2) We would need several kg of HMOs to administer to the intervention group every 2 to 3 h for at least the first four weeks of life, and HMOs were simply not available in that amount. (3) There was no information which of the more than 150 different HMOs would be effective. It could be that all HMOs are effective, but it could also be that the effects are highly structure-specific and limited to just one or two selective HMOs. (4) The study design itself was (and remains to be) challenging. It is known that formula-fed infants are at a significantly higher NEC risk and it would be unethical to use formula-feeding without HMOs as intervention control. Thus, we selected a rodent NEC model to test our hypothesis first, allowing us to use much smaller amounts of HMOs for initial efficacy testing. Afterwards, the small animal model would also enable us to conduct structure-activity relationship (SAR) studies and elucidate the underlying mechanisms of action.

The NEC model in neonatal rats was originally described by Barlow and Santulli (47) and later modified as follows (48): Pregnant time-dated Sprague-Dawley rats were induced at term. The pups were immediately removed from the dam at birth to ensure they don't receive any rat milk, which also contains some oligosaccharides. The pups were randomized into one of the different study groups. Some pups were returned to the dam to serve as dam-fed control. All other animals remained separated from the dam, housed in a temperature- and humidity-controlled incubator and, twice daily, orally gavaged with a special rodent formula with or without HMOs that were isolated from pooled human donor milk. All animals, dam-fed and gavaged, were exposed to 10 min of hypoxia thrice daily in a modular chamber. All animals were sacrificed 96 h post-partum; their intestines were collected and inspected for the presence of gross necrotic changes or NEC-characteristic *Pneumatosis intestinalis*. A section of the terminal ileum was prepared for H&E staining and scored blindly based on morphological changes that included epithelial sloughing, villus oedema, infiltration of neutrophils, apoptosis of villus enterocytes, crypt hyperplasia, and misaligned nuclei in the epithelium.

The HMO intervention had an immense effect in the neonatal rat NEC model. Pups that received HMOs with their formula had a significantly higher survival rate than their littermates that did not receive HMOs (49). Their intestines were not as dark and bloody with less patchy necrosis and less evidence of haemorrhagic intestine as well as intramural gas cysts (*Pneumatosis intestinalis*). These macroscopic observations were aligned with microscopic evaluation of ileum sections, showing that pups that received HMOs had significantly lower pathology scores than their littermates that did not receive HMOs. From this first set of experiments we concluded that HMOs indeed reduce NEC-like symptoms in the neonatal rat model and even improved survival significantly.

## A Specific HMO, Disialyllacto-N-Tetraose (DSLNT) Is Most Effective in Reducing NEC in Neonatal Rats, But the Underlying Mechanisms Are Likely Different Than Originally Postulated

**Figure 3:**
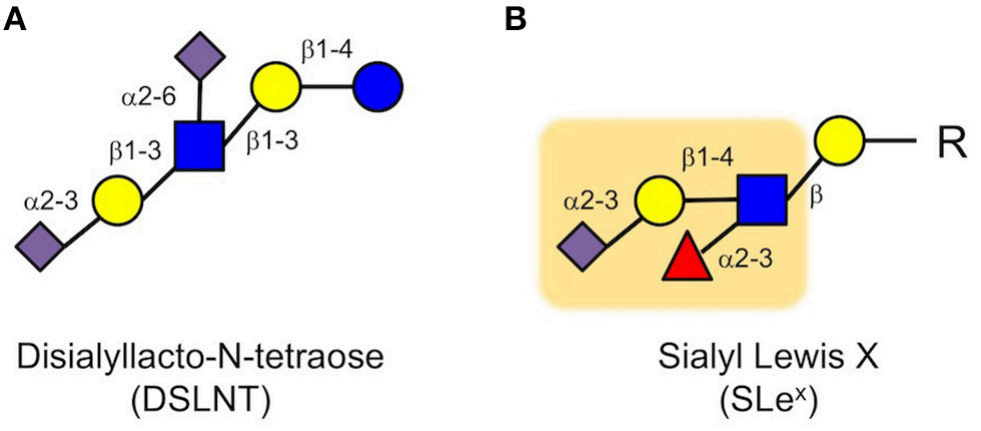
Disialyl-lacto-N-tetraose (DSLNT) **(A)** reduces NEC-like symptoms in neonatal rats, but the HMO does not contain the Sialyl Lewis X (SLe^x^) determinant **(B)**. The Sialyl Lewis X is highlighted as an orange background (blue circle, glucose; yellow circle, galactose; blue square, N-acetylglucosamine; red triangle, fucose; purple diamond, sialic acid).

Next, we applied a multidimensional chromatography approach to address the question which of the more than 150 different HMOs was responsible for the beneficial effects we observed in the neonatal rat model (49). In a first dimension, we used anion exchange chromatography to separate the HMOs by charge. Every sialic acid moiety contributes a negative charge to an HMO. HMOs without sialic acid carry no negative charge and are neutral; HMOs with one sialic acid carry one negative charge; HMOs with two sialic acids carry two negative charges, etc. We then tested these charge-fractions in the neonatal rat NEC model and found that HMOs with two sialic acids were most effective. However, there are several HMOs with two sialic acids. Thus, in a second dimension, we used gel filtration chromatography to separate the fraction that contained HMOs with two sialic acids by size. We then tested the generated subfractions for their efficacy in the neonatal rat NEC model and identified one specific subfraction, containing only one HMO, that was most effective. Finally, we used a combination of exoglycosidase enzyme digestion and gas chromatography mass spectrometry (GC-MS) of partially methylated alditol acetate (PMAA) derivatives to structurally characterize the effective HMO as disialyllacto-N-tetraose (DSLNT, Figure 3A). We further confirmed that the effect of DSLNT is highly structure-specific. For example, enzymatic removal of sialic acid at the terminal galactose completely abolished the effect.

While these results were very exciting, they were also quite puzzling. The *in vitro* and *ex vivo* data showed that HMOs interfere with selectin-mediated cell-cell interactions, leading to a reduction in neutrophil rolling, adhesion and transmigration as well as a reduction in neutrophil activation, which were considered key elements in NEC pathogenesis. However, we did not observe a reduction in neutrophil infiltration in the neonatal rat NEC model. Moreover, DSLNT, the HMO we identified as being most effective in reducing NEC-like symptoms in rats, did not contain a SLe^x^ determinant that is part of selectin ligands (Figure 3). While there is some structural ambiguity around the glycan determinant for selectin ligands (50), fucose is an essential component for a glycan to serve as a selectin ligand as demonstrated in patients with congenital disorder of glycosylation (CDG) type IIc, also known as leukocyte adhesion deficiency (LAD) type 2 (51). The genetic defect in CDG IIc patients leads to impaired intracellular fucose metabolism, which results in decreased fucosylation of cell surface proteins (52), including selectin ligands. Consequently, CDG IIc patients present with impaired neutrophil motility and extravasation and recurrent infections (51). Although DSLNT is effective in reducing NEC-like symptoms in the neonatal rat model, it is not fucosylated. Thus, it appears unlikely that DSLNT interferes with selectin-mediated neutrophil infiltration and activation. Therefore, while the hypothesis that HMOs contribute to a lower NEC risk in human milk-fed infants may indeed be correct, the underlying protective mechanisms are likely different than originally anticipated.

While it is known that HMOs shape microbial communities (26–28, 31), it remains challenging to establish direct cause-and-effect relationships. There are at least two different scenarios to connect DSLNT, the microbiome, and NEC-like symptom improvement: (1) DSLNT affects the microbiome which then affects the host and improves NEC-like symptoms, and (2) DSLNT affects the host, and the host response leads to an improvement in NEC-like symptoms and also, and independently, to a change in microbiome.

In addition to influencing the microbiome and targeting a NEC-associated dysbiosis, HMOs can also alter host epithelial cell or host immune cell responses. These interactions are often receptor-mediated and highly structure-dependent, which would explain why DSLNT is effective, but the removal of just one sialic acid moiety from DSLNT renders the HMO ineffective. While selectins require their glycan binding partners to be fucosylated and DSLNT is not fucosylated, other glycan-binding receptors like galectins or siglecs play major roles in facilitating and modulating immune responses and represent potential DSLNT targets (53).

We have since explored the chemical space around DSLNT and tested *in vivo* efficacy of chemoenzymatically synthesized derivatives in the neonatal rat NEC model (54–56). Interestingly, the HMO 2′-fucosyllactose (2′FL), which is structurally unrelated to DSLNT, available at commercial scale, and now added to some term infant formula, had a moderate effect in the neonatal rat and mouse NEC models (55, 57), but failed to improve NEC in a piglet model (58). So far, DSLNT remains to be the most effective HMO or derivative we have studied in the context of NEC to date.

## Human Mother-Infant Cohort Studies Confirm That DSLNT Is Associated With Lower NEC Risk

While the data obtained from HMO efficacy testing in the neonatal rat NEC model are encouraging, the use of preclinical NEC models in rodents or piglets is challenging (59). Animals are exposed to rather artificial insults like external hypoxia and/or hypothermia. In general, the use of animals itself is considered a major limitation due to interspecies differences in anatomy, physiology, and pathophysiology. Therefore, advancing a potential therapeutic like DSLNT from preclinical models, that are controversial, to clinical treatment trials, that are challenging and expensive, comes with a tremendous risk of failure. To narrow this wide gap between preclinical models and clinical intervention studies, we applied an intermediate approach and conducted a prospective cohort study with mothers and their very low-birth-weight (VLBW) infants fed predominantly human milk (60). The study is based on the observation that some of the infants who receive predominately human milk still develop NEC. However, not all human milk is equal. In fact, there is strong interindividual variation in HMO composition, which led us to hypothesize that human milk fed to infants who develop NEC contains less DSLNT than human milk fed to infants who do not develop NEC.

The study was conducted in five different neonatal intensive care units across North America (US and Canada), recruited 200 mothers, and analyzed HMO composition in human milk fed to their VLBW infants over the first 28 days post partum (60). We then matched each of the eight identified NEC cases with five controls, and used logistic regression and generalized estimating equation to show that DSLNT concentrations were significantly lower in almost all milk samples in all eight NEC cases when compared to controls. The association between low DSLNT concentrations in human milk and NEC was highly significant (*p* < 0.001), corroborating the results that DSLNT reduces NEC-like symptoms and improves survival in neonatal rats (49).

In parallel, we analyzed the HMO composition in human milk samples from a mother-infant cohort in South Africa, and found overlapping results (61). Although the original and primary objective of the South Africa study was to investigate the role of HMOs in reducing HIV-transmission in preterm infants, the cohort included several VLBW infants who developed NEC. Independent of the HIV-status, DSLNT concentrations in the milk that these infants with NEC received were significantly lower than those in the milk given to infants who did not develop NEC. Thus, data from two independent cohort studies, one in North America and one in South Africa, strongly support our hypothesis that a specific HMO, DSLNT, contributes to the lower NEC risk in human milk-fed infants.

As the two cohort studies have shown, DSLNT concentrations vary greatly between women with preterm infants, but seem to be fairly constant within the same woman over the first four weeks of lactation (60, 61). The variability in DSLNT concentrations between different donors may be one justification for donor milk to be pooled or synthetic DSLNT to be added. Like other HMOs, DSLNT concentrations are not affected by pasteurization (62–64). However, when comparing HMO composition in donor milk batches from the San Jose Milk Bank with that in milk from moms with preterm infants in the neonatal intensive care unit at the University of California, San Diego, we discovered that DSLNT is slightly lower in donor milk batches, potentially reflecting the fact that donor milk is often from women with healthy term infants, which might be different from milk of women with preterm infants (63).

## Future Perspective

**Figure 4:**
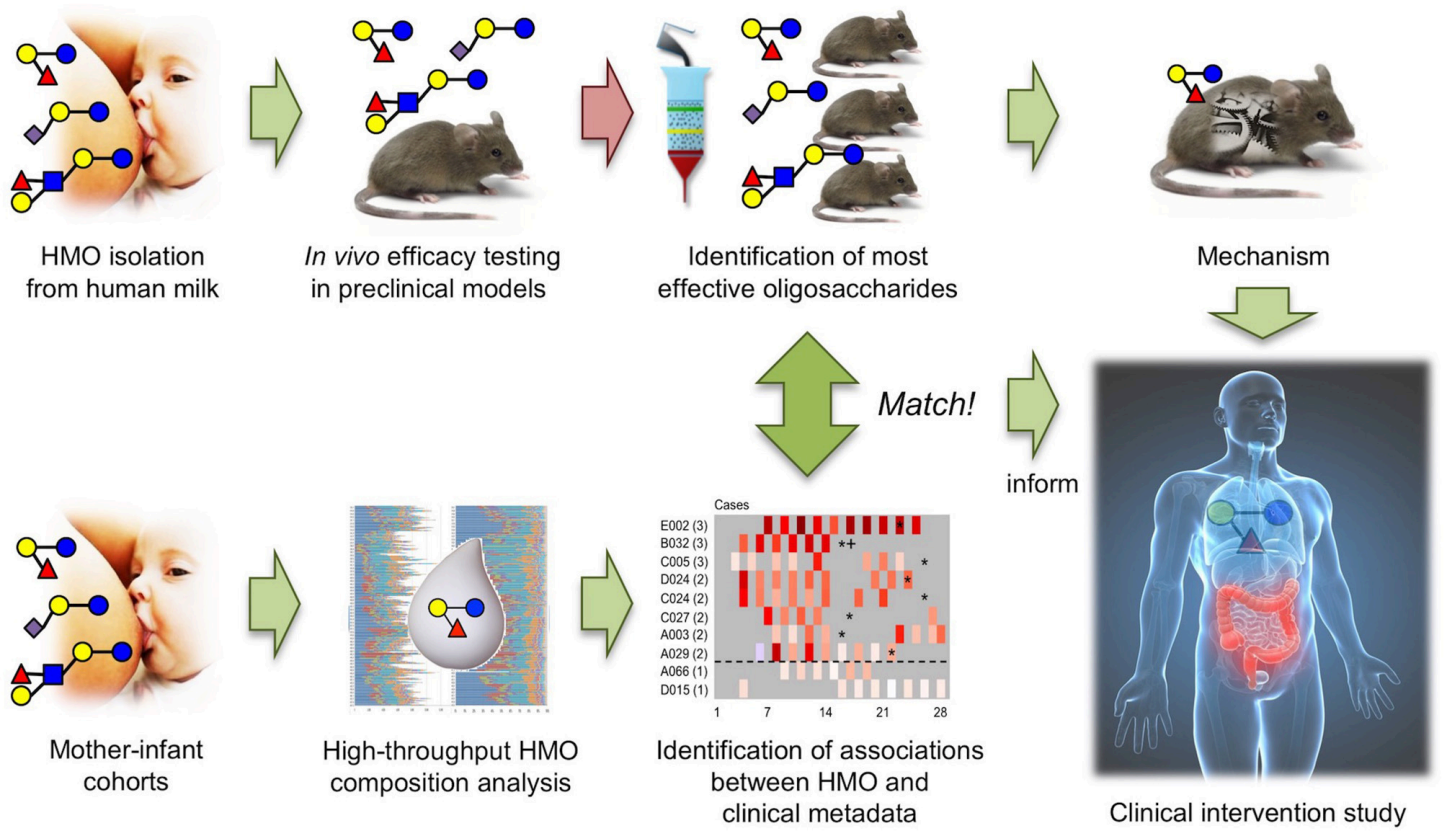
The combination of *in vivo* HMO efficacy and structure-function testing in neonatal rats with association studies in human mother-infant cohorts led to the identification of DSLNT as the protective HMO in NEC and informs future invention studies.

The results from the North American and South African cohort studies match the results from *in vivo* efficacy testing and structure-activity relationship studies in the neonatal rat NEC model, providing a strong foundation to further explore DSLNT as a therapeutic for NEC and setting a powerful example of how the combination of *in vitro*/*ex vivo, in vivo*, and cohort studies can advance a field (Figure 4).

Even though some of the control milk samples in the North American cohort occasionally had low levels of DSLNT concentrations, the aggregate assessment of DSLNT in milk fed to the same infant over multiple days greatly increased the ability to discriminate between NEC cases and controls (60). Therefore, in addition to exploring DSLNT as a new NEC therapeutic, measuring DSLNT content in mother's own milk has the potential to serve as a non-invasive marker to identify infants at risk of developing NEC. Furthermore, DSLNT could become a quality control parameter for donor milk and products like human milk-based human milk fortifiers to avoid feeding low DSLNT products to infants at risk to develop NEC. Although other HMOs like 2′FL or lacto-N-(neo)tetraose have been successfully synthesized at large scale and are currently added to some formula for healthy term infants, the synthesis of DSLNT remains to be challenging and is not yet available for preterm infants at risk of NEC.

## Author Contributions

The author confirms being the sole contributor of this work and has approved it for publication.

### Conflict of Interest Statement

The author declares that the research was conducted in the absence of any commercial or financial relationships that could be construed as a potential conflict of interest.
